# Interactions Between Allogeneic Mesenchymal Stromal Cells and the Recipient Immune System: A Comparative Review With Relevance to Equine Outcomes

**DOI:** 10.3389/fvets.2020.617647

**Published:** 2021-01-13

**Authors:** J. Lacy Kamm, Christopher B. Riley, Natalie Parlane, Erica K Gee, C. Wayne McIlwraith

**Affiliations:** ^1^School of Veterinary Science, Massey University, Palmerston North, New Zealand; ^2^Hopkirk Laboratory, AgResearch, Palmerston North, New Zealand; ^3^Orthopaedic Research Center, C. Wayne McIlwraith Translational Medical Institute, Colorado State University, Fort Collins, CO, United States

**Keywords:** allogeneic, mesenchymal stromal cell, equine, immune, lymphocyte

## Abstract

Despite significant immunosuppressive activity, allogeneic mesenchymal stromal cells (MSCs) carry an inherent risk of immune rejection when transferred into a recipient. In naïve recipients, this immune response is initially driven by the innate immune system, an immediate reaction to the foreign cells, and later, the adaptive immune system, a delayed response that causes cell death due to recognition of specific alloantigens by host cells and antibodies. This review describes the actions of MSCs to both suppress and activate the different arms of the immune system. We then review the survival and effectiveness of the currently used allogeneic MSC treatments.

## Introduction

Bone marrow-derived mesenchymal stromal cells (MSCs) possess immense potential for the treatment of many diseases (1, 2), and there has been rapid acceleration in the clinical use of MSCs (2). Bone marrow-derived MSCs have become the “gold standard” MSC for use in musculoskeletal therapies (3, 4), though adipose-derived and umbilical cord-derived MSC products are also commonly available (3). The use of allogeneic MSCs for treatment is less costly as it can be prepared for multiple animals and is immediately available for treatment (5, 6). An additional benefit is for older patients whose MSCs are known to have lower proliferation rates as compared to MSCs from younger donors (7). Allogeneic MSCs as an off the shelf product will likely be the main mode of MSC treatment in the future.

When treating an individual, be it human or equine, with allogeneic stem cell therapy, prevention of allorecognition of the recipient to the transplanted foreign antigens is an important component of achieving a persistent and potent effect. Medium- or long-term survival of the MSCs to exert their desired anabolic effects is likely to promote their effectiveness as a treatment as compared responses associated with short-term survival. Certainly, short-lived therapy with MSCs can be the catalyst for improvement of disease processes, as several studies have reported that the number of implanted MSCs detected in target tissue was too low to explain the improvement in disease state (1). However, without survival of the MSCs, there would be no source of ongoing therapeutic effect nor involvement of the MSC in the structural integrity of repair. Another concern regarding allorecognition is the described side effects of intra-articular allogeneic MSC injection in people (5) and horses (8). These include pain, swelling of the joint, and urticarial (5, 8). For these reasons, a complete understanding of the interaction of the MSCs with the immune system is necessary to foresee the risks and predict the effectiveness of allogeneic MSCs as a treatment.

Many studies have found that bone-marrow-derived MSCs are capable of substantial anti-inflammatory effects (9–13). The immunomodulation caused by MSCs is dependent on inhibitory molecule secretion, direct cell contact, and induction of regulatory leukocyte populations (14–17). Over 350 human studies are currently underway that investigate the ability of MSCs to limit immune reactions related to auto-immunity and tissue transplantation (18). Previous studies have shown that allogeneic MSCs suppress immune reactions in graft-vs.-host disease and organ transplantation even when steroids are unable to provide suppression (19).

From understanding the published literature, we know that allogeneic MSCs have both immunostimulatory and also immunosuppressive actions. What we must determine is the overall effect. Are allogeneic MSCs used in the equine patient able to provide anti-inflammatory and anabolic effects or does immune recognition negate these therapeutic benefits?

## The Interaction of the Innate Immune System With Allogeneic MSCs

The cascade of events that occurs when MSCs encounter the immune system can be broken down into phases of the immune system response. These include the acute reaction by the innate immune system, and then the slightly delayed specific adaptive immunity of both cell-mediated and humoral (antibody) responses that result in long-term memory cells (20). It is important to understand how the MSCs are affected through each of these steps in order to determine the potential for efficacy and the side effects of allogeneic treatment.

The innate immune system responds quickly and non-specifically to foreign antigens. This involves the release of anti-microbial enzymes and peptides, complement activation, recruitment of inflammatory cells, phagocytosis and destruction of foreign pathogens, and cells (20). Endothelial cells are one of the first cells to detect foreign pathogens, resulting in release of chemokines which allow the blood vessels to dilate leading to the extravasation and migration of phagocytes such as neutrophils and macrophages (20).

### Complement

The complement system is an important part of innate immunity. Complement is released from the liver into the blood in its inactive form and is cleaved to create its activated form by proteases derived from inflammation. Complement components can bind directly to alloantigens or utilize antibodies to mark the antigen for removal (21). The foreign cell is then removed by forming a membrane attack complex or by facilitating leukocyte phagocytosis (21, 22).

When the effects of complement are considered alone without accounting for the actions of other immune cells, this non-cellular agent has been shown to cause a decrease in viability of human allogeneic MSCs (22–24). Two studies found >40% of human adipose-derived MSCs were damaged upon culture with naïve human serum containing activated complement (23, 24). Another study found minimal damage to MSCs when complement alone was added, but complement-mediated phagocytosis caused MSC death when monocytes were added *in vitro* (22). Means of resolving complement-mediated cytotoxicity have been created, but thus far each requires manipulation of the MSCs by means of application of complement-inhibiting materials (Factor H or N-glycolylneuraminic acid) to the cells' surface which is likely impractical from a licensing perspective at this point in time (24, 25). CD59, a molecule found on some MSCs can prevent complement opsonization (22). Sourcing MSCs with high surface expression of CD59 may also be a potential means to mitigate complement-mediated MSC death (22).

The effects of the complement system on equine MSCs have not yet been reported in the horse.

### Neutrophils

Neutrophils are the most numerous cell of the innate response and often the first leukocyte to infiltrate an allogeneic tissue (20, 26). Neutrophils are recruited to areas of inflammation by vascular endothelium and likely recruited to MSCs by chemokine proteins such as CXCL8 (IL-8) (26, 27). Once extravasated into allogeneic tissue, neutrophil infiltration leads to increased antigenicity and reduced allograft function (28). This may not occur when MSCs are administered as MSCs cause minimal activation of neutrophils *in vivo* by allogeneic MSCs (29). Allogeneic MSCs appear to be immunomodulatory in that they can suppress neutrophil activation by causing a significant reduction in ROS when neutrophils were activated prior to the addition of MSCs (28–31).

Although neutrophils in isolation are not activated by MSCs, one of the most concerning effects of the innate immune system in the horse is the rapid influx of neutrophils following intra-articular (both autologous and allogeneic) MSC injection (8, 32). Numerous studies investigating the effect of MSC injection into equine joints show an increase in neutrophil count in synovial fluid lasting 48–72 h after administration of autologous and allogeneic stem cells (8, 32–34). An increase in effusion (as measured by joint circumference) with or without a mild increase in lameness also occurs at similar time points (8, 32–34). There are several confounding factors for this neutrophil invasion. Joswig et al. (32) showed this increase in cell infiltration and swelling occurs to the same degree when MSC freeze media (autologous serum and 5% DMSO) is injected alone without MSCs, as when freeze media is injected with autologous or allogeneic MSCs. The authors determined that in these cases, MSCs may not be the primary cause of neutrophil infiltration (32). Another contributor to neutrophil activation found in earlier studies is the use of FBS in MSC media (32). There is a significant increase in nucleated cell counts in the synovial fluid of joints injected with FBS-cultured autologous MSCs as compared to autologous or allogeneic MSCs cultured in equine serum during the final 48 h of incubation (32). Because of this finding, where possible, studies are performed without this confounding factor.

Another possible cause of neutrophil influx may be due to a small proportion of MSCs in a cryopreserved or fresh MSC sample that become non-viable prior to administration (35). Activated neutrophils participate in the clearance of apoptotic cells; therefore, neutrophils enter the joint following an injection of dead cells. Interestingly, because apoptotic cells inhibit the proinflammatory functions of neutrophils, uptake of apoptotic cells by neutrophils can contribute to the resolution of inflammation in areas where dead cells are present (36). The degree to which dead MSCs cause neutrophil influx as compared to live MSCs is unknown.

In a different type of study, MSCs had immunosuppressive effects on neutrophils in an inflamed equine joint (37). In this study lipopolysaccharide (LPS) was injected into one joint to stimulate an inflammatory response, and LPS and umbilical cord-derived MSCs were injected into the contralateral joint. This study saw a significant decrease in neutrophil influx into the joint after injection of both MSCs and LPS compared to the injection of LPS alone (37). The interpretation of these findings is that the presence of MSCs suppresses the activation of innate immune system.

Overall, there is concern when a horse is treated with either autologous or allogeneic MSCs and the joint then becomes acutely swollen and/or lame. In layman's terms this reaction is called a “flare”; a short-lived inflammatory response that resolves without treatment or with anti-inflammatory medication. Flares in clinical cases have been reported to occur in between 1.8 and 9% of equine cases receiving autologous or allogeneic MSCs (38, 39). No long-term negative effects were seen in either of these studies. Human studies using allogenic MSCs and hyaluronic acid had a 25–53% rate of significant effusion after intra-articular treatment of the knee (40, 41), while administration of autologous MSCs and hyaluronic acid had a 45% rate of effusion (42). When, hyaluronic acid was used alone, 60% of human patients suffer from significant effusion (40).

Although these brief incidents of soreness and swelling can be worrying to the client, there is no evidence of long-term negative effects nor lack of response to treatment (39, 40). Additionally, as laboratories replace FBS during the final 48 h of culture, these “flares” should be less common. Therefore, neutrophil influx after allogeneic MSC treatment in the horse does not appear to be an impediment to the use of allogeneic MSCs.

### Macrophages

Macrophages are the most efficient type of phagocyte and are able to eliminate a large variety of pathogens, including foreign cells (43). When human allogeneic MSCs are cultured with macrophages, the macrophages become immunosuppressive, inhibiting natural killer (NK) cells and pushing T lymphocytes down a regulatory pathway (44). At this time there are only two equine studies that have reported the reciprocal effects of MSCs and macrophages. Cassano et al. (45) found minimal effect of MSCs on activated macrophages *in vitro* showing that MSCs may not have a strong immunoregulatory ability to deactivate macrophages. Those MSC exposed to activated macrophages, though, then became immunosuppressive in an activated T lymphocyte proliferation assay (46). Although data in this area are extremely limited, allogeneic MSCs may be less capable of immunomodulation of activated macrophages (45).

### Natural Killer Cells

Natural killer cells are a part of the innate immune system that can cause cell death through the targeted release of cytotoxins (47). NK cells can attack cells lacking major histocompatibility complex (MHC) I on the surface of cells (47). As bone marrow-derived equine MSCs express MHC I (48, 49), NK cells may be less likely to pose a threat for these MSCs. Any hypothesizing on this issue is debatable at this point as appropriate antibodies for recognition of NK cells in the horse are lacking. MSCs have been found capable of suppressing NK cytotoxic activity in a murine hepatotoxicity model and using human cells *in vitro* (50, 51).

### Dendritic Cells

Dendritic cells capture and process alloantigens and serve to activate the adaptive immune system by presenting the alloantigens to B and T lymphocytes (52). Dendritic cells cultured with murine allogeneic MSCs cause the dendritic cells to decrease their surface expression of stimulatory molecules including CD80, CD83, CD86, and MHC II (53). In response to pathogens, these molecules are normally up-regulated to aid in activation of cell-mediated immunity. After interaction of the dendritic cells with murine allogeneic MSCs, the dendritic cells then cause a decrease in lymphocyte proliferation in mixed lymphocyte reactions (53). Here we see evidence of the inhibition of adaptive immune system through MSC effects on the innate responses.

## The Interaction of the Adaptive Immune System With Allogeneic MSCs

As previously mentioned, the adaptive immune response consists of two primary pathways; one is cell-mediated and the other is antibody-mediated (i.e., humoral immunity). T lymphocytes are needed for both pathways. In the humoral response of the adaptive immune system, B cells or antigen presenting cells bound with alloantigens in association with major histocompatibility type II (MHC II) receptor interact with helper T cells (i.e., CD4 T lymphocytes) (54, 55). Upon interaction with CD4 lymphocytes, B cells then are activated to differentiate into plasma cells which secrete antibodies to the alloantigen (55). The earliest antibodies are seen in circulation after invasion of the organism is just less than 1 week (48, 56), and these antibodies can circulate for a long duration (56, 57). This may be important in clinical scenarios where repeat treatments with allogeneic equine MSCs are warranted.

The cell-mediated component of the adaptive immune response requires cytotoxic T cells (i.e., CD8 T lymphocytes). Cytotoxic T cells take part in both direct and indirect alloimmunity with cells bearing MHC I receptors that are bound with an alloantigen. In this way, cytotoxic T cells attack those cells that are foreign to the organism or cells that have taken up a foreign antigen. After a pathogen is recognized, a subset of CD8 cytotoxic T cells mature to form memory T cells (58). Memory T cells rapidly respond upon subsequent antigen recognition, triggering the removal of the foreign antigens even many years later (58). Both CD4+ and CD8+ lymphocytes are important when considering the use of allogeneic MSCs as these immune cells may recognize allogeneic MSCs due to their expression of MHC I and II.

### MHC I and II Expression on MSCs

After some debate about the presence of major histocompatibility markers on equine MSCs, it is now known that the cell surface expression of MHC I and II on MSCs is variable from one donor to another and even one MSC sample to another (48, 49, 59). MHC I is expressed on all equine bone marrow-derived MSCs though the degree of expression varies (48). Conversely, some MSCs do not express MHC II antigens, while others have a strong positive expression (49, 59). Most problematically, MHC I and II expression is increased in the face of culture with foreign lymphocytes, when MSCs are cultured with inflammatory cytokines, or as the MSCs differentiate (46, 60–62). The expression of MHC I and II motifs on MSCs are important in that they are the key cell markers utilized for alloimmunity by the host's immune system, and expression of these markers identifies the MSCs as targets for destruction. Not only is the expression of these molecules important, but the degree to which these molecules are similar between the donor and recipient is also critical. The structure of each MHC molecule is defined by the human leukocyte antigen (HLA) or equine leukocyte antigen (ELA) haplotype (63). Horses are haplotyped using microsatellites to the ELA gene (63). The ELA haplotype and degree of mis-matching determines the recognizability of donor cell to the recipient's immune system. Therefore, an MSC that expresses MHC I or II would be minimally immunogenic if the ELA haplotype is “matched” to the recipient (61, 64).

### T Lymphocyte Responses to MSCs

What is the overlying result when allogeneic MSCs are exposed to lymphocytes? Are the lymphocytes activated or suppressed? When suppression of activated lymphocytes is considered, studies have overwhelmingly shown that allogeneic equine MSCs are capable of preventing lymphocyte proliferation in response to an activating agent (phytohaemaglutinin, foreign leukocytes, etc), thereby quelling an immune response (10, 11, 13, 65). This immunosuppression occurs subsequent to the MSC-mediated increase in regulatory T lymphocytes (Tregs) which serve to dampen the adaptive immune response and can prevent rejection of foreign cells by the host (66). MSCs secrete immunomodulatory cytokines, including transforming growth factor beta (TGF-β), indoleamine 2,3-deoxygenase 1, IL-2, IL-10, IL-1beta receptor antagonist, hepatocyte growth factor and PGE2 (11, 67–70). These cytokines serve to push the T lymphocytes down the path to create more T regulatory cells and to suppress leukocyte activation (11, 70).

Many *in vitro* studies have been performed looking into lymphocyte behavior after interaction with MSCs. Two studies using equine MSCs, showed that both autologous and allogeneic MSCs have an equal immunosuppressive capacity when MSCs are cultured with activated lymphocytes (11, 13). This may indicate that immunosuppression is the predominant response when compared with immunoactivation by allogeneic MSCs. Another study examined activated lymphocytes and how they interacted with different types of allogeneic equine MSCs (59). Suppression of the lymphocytes occurred when MSCs expressing low levels of MHC II were co-cultured, but increased activation occurred when MSCs expressing high levels of MHC II were co-cultured (59). A study using 11 different human allogeneic MSC products found that every product tested was capable of immunosuppression when cultured with activated lymphocytes (65). These studies indicate allogeneic MSCs are repeatedly shown to be capable of suppressing activated T lymphocytes. It must be acknowledged that each of these studies were performed *in vitro*, and previous studies in the horse have shown a lack of correlation in immunomodulatory properties between *in vitro* and *in vivo* results (59, 71).

Do allogeneic MSCs cause activation of unactivated lymphocytes? Colbath et al. (11) has shown that allogeneic and autologous equine MSCs cause mild lymphocyte proliferation *in vitro*, the extent of which was similar for both groups. Similarly, in humans, lymphocyte proliferation occurs when lymphocytes are co-cultured with allogeneic MSCs (72). Interestingly, several human studies found an immunosuppressive form of the MHC I antigen, called HLA-G, which is expressed on some human MSCs (9, 72, 73). Nasef et al. (73) found that by adding an antibody against HLA-G, effectively inhibiting it from performing its function, activated lymphocytes proliferate when mixed with allogeneic MSCs. Without the neutralizing antibody, human allogeneic MSCs prevent lymphocyte activation. Other work has shown HLA-G causes lymphocyte suppression and increases the number of immunosuppressive Tregs (9). This HLA-G form of the MHC I molecule, which provides an innate ability to prevent the recognition of foreign cells, has likely evolved from the need to prevent fetal attack during gestation (9, 73). This immunosuppressive isoform of MHC I is likely to exist in the ELA system, though no evidence has yet been published for the horse.

Does repeat exposure of the T lymphocytes to an allogeneic MSC cause lymphocyte activation? Piggot et al. (74) co-cultured allogeneic MSCs with lymphocytes from horses that had previous exposure to the allogeneic MSCs and found no CD4+ lymphocyte proliferation signifying a lack of CD4+ memory cells. Koi et al. (75) found that the systemic CD8+ population of lymphocytes, not the CD 4+ lymphocytes, increased when horses were treated for a second time with intravenous allogeneic MSCs. This suggests that CD8+ memory T cells are generated upon original exposure leading to cytotoxic lymphocyte proliferation upon re-injection with MSCs (75).

### B Cells and Alloantibody Responses to MSCs

Antibody production has been shown to be a limitation for allogeneic MSC survival. There is significant antibody production to allogeneic MSCs across species (71, 76, 77). Barrachina et al. (62) found that all equine patients receiving intra-articular allogeneic mis-matched MSCs formed antibodies after injection. Pezzanite et al. (71) used MSCs of a mis-matched ELA haplotype and injected these cells intradermally in horses. After 21 days, all horses had synthesized antibodies against the ELA type of the MSC that had been administered (71). These antibodies are capable of targeting the MSCs for destruction (64). Of the six horses tested, one also created an antibody response to another ELA type (71). This cross reactivity has been reported previously in the human literature (78, 79).

The synthesis of antibodies capable of destruction of the MSCs after allogeneic treatment may limit the survival of the MSCs and therefore decrease the potency of therapeutic effect. Overcoming the undesirable consequences of the adaptive immune response is important when repeat MSC treatment is required as antibodies to the MSC may be present on administration (59). There are several methods to mitigate alloantibody production. One way forward is to ELA type donors and recipients to find a “matched” pair. This is challenging as there are at least 50 variations in ELA haplotypes (63). Another strategy is to ELA type the donor horses of the MSCs and give subsequent treatments with MSCs of a different haplotype. Using this technique, only the horses that have cross-reactive antibodies would carry antibodies against the MSCs at the time of treatment. A third possible technique relies upon the manipulation the MSCs to prevent expression of MHC I and II. The reduction of MHC I and II expression has been successfully performed in human and murine MSCs using molecular biologic techniques (6, 80). The addition of TGF β2 has also been shown to reduce MHC I and II expression (48).

Even without these techniques to decrease the effects of the major histocompatibility molecules, the MSCs that are currently being utilized provide beneficial treatment effects despite alloimmunity being present (38, 81–86).

## Allogeneic MSC Survival *in vivo*

There is some controversy as to whether there is a considerable beneficial effect of longer-term MSC survival in damaged tissue as compared to a short-lived effect. One study found that dead MSCs used to treat cardiac ischemia-reperfusion injury in mice had the same beneficial effect as viable MSCs (87). This study determined that the effect of MSCs on macrophages caused the improvement in cardiac output. Another study with the same method of cardiac insult found a significant effect between MSC survival and improved cardiac function (88). The MSCs in this second study were tracked over 30 days and were found to be present in the myocardium throughout the study period. These studies seem to conflict with one another, but perhaps this is due to the method of improvement in function seen in the different studies. An immune-mediated effect may not necessitate long term MSC survival as some reports suggest (1, 88, 89), while a structural effect may require long-term MSC incorporation.

Few equine studies focusing on the duration of survival of allogeneic MSCs have yet been published. Furthermore, it is largely unknown what percent of the original dose of MSCs that is given to a patient survives long term, but generally this is believed to be a very small proportion for both autologous and allogeneic MSCs (90, 91). Guest et al. (90, 91) found that ~2% of the originally injected equine bone marrow-derived allogeneic MSCs survived to 30 days in the lesion and 1% survived to 60 days in the lesion (Table 1). Ovine bone marrow-derived allogeneic MSCs survive at least 6 weeks after intra-tendinous injection though the percent survival was not measured (Table 1) (92). Human MSCs injected into mice survive longer than 5 months when injected intramuscularly, 1–4 weeks when injected subcutaneously or intraperitoneally, but only a few days when injected intravenously (Table 1) (93). When allogenic adipose-derived MSCs were used intra-articularly after disease induction in the femorotibial joint, MSCs survived 10 weeks in the rat and 14 weeks in sheep (Table 1) (94, 95). By extrapolating the data in these studies, it appears that allogeneic MSCs survive for a longer period in areas of lower vascularity.

**Table 1:** Relevant allogeneic MSC survival studies.

**Study**	**Recipient specie, Tissue treated, number of cases**	**MSC origin**	**Survival measurement method**	**Survival duration (days)**
Guest et al. (90, 91)	Equine, experimental tendon lesion, *n* = 8	Equine bone marrow	Green fluorescent protein (GFP)	<5% of MSCs survive past 10 days, present in lesion >60 days, no difference between allogeneic and autologous
Lactignola et al. (92)	Ovine, Achilles tendon, *n* = 9	Ovine bone marrow	Red florescent protein	>6 weeks (all allogeneic)
Braid et al. (93)	Murine, *n* = 3–5/ location	Human bone marrow or umbilical cord	Luciferase lentivirus	>110 days intramuscular, 7 days subcutaneous, 21 days intraperitoneal, 3 days intravenous
Li et al. (94)	Murine, intra-articular, *n* = 3 at each time point	Human adipose	DiD fluorescent dye	2/3 rats at 14 days and 1/3 rats at 70 days
Feng et al. (95)	Ovine, *n* = 24	Human adipose	Iron visualization via MRI	All sheep at 98 days

## Results of Allogeneic MSC Therapy for Musculoskeletal Disease

Above and beyond the possible mechanisms for deleterious effects on MSCs by the immune system, the results of *in vivo* clinical trials and experimental studies must be considered. The use of bone marrow-derived allogeneic MSCs for joint disease has gained popularity, likely due to largely positive results (2, 96). A large equine clinical trial of 165 horses treated with allogeneic MSCs and platelet rich plasma has been described (38). In this report 45% of cases at 6 weeks post-treatment, and 78% of cases by 18 weeks returned to athleticism, though this study lacked a control population (Table 2) (38). A study using a chemically induced- model of arthritis in the horse showed significant upregulation of type 2 collagen and significantly decreased expression of inflammatory mediators in cartilage at 6 months post-treatment when allogeneic MSC-treated joints were compared to untreated joints, though no significant gross nor histologic improvement was seen (Table 2) (33). In a similar study, allogeneic MSCs did not cause significant clinical improvement in IL-1beta- induced arthritis, however, this was a very acute and severe inflammatory model (Table 2) (97). Additional allogeneic MSC studies focusing on joint disease in the horse have shown beneficial clinical and histologic results using blood-derived (81), neonatal-derived (82), or adipose-derived MSCs (Table 2) (83). In people with severe knee osteoarthritis, Vega et al. (40) showed improved function and cartilage grade on MRI as compared to hyaluronic acid when MSCs were used intra-articularly. Experimentally created knee arthritis in rabbits was improved when treated intra-articularly, but only when the animals were treated on three occasions as one injection was insufficient to improve outcomes (98). The use of allogeneic MSCs in joint disease appears to be beneficial though in some studies, this benefit was not clinically relevant.

**Table 2:** Relevant equine studies evaluating the use of allogeneic MSCs in clinical and experimental musculoskeletal disease.

**Study**	**Type of MSC used**	**Disease treated, number of cases**	**Negative effects?**	**Positive effects?**
Broecx et al. (38)	MSC from peripheral blood or chondrogenic induced MSC	Clinical osteoarthritis, *n* = 165	Flare in 1.8% of 165 horses	78% return to athleticism for native MSCs and 86% for chondrogenic induced MSCs
Broecx et al. (81)	Chondrogenic induced MSC	Clinical metacarpophalangeal osteoarthritis, *n* = 75	No	Significant improvement in lameness, flexion, joint effusion score by 18 weeks post-injection
Barrachina et al. (33)	Bone marrow-derived MSCs	Chemically induced arthritis, *n* = 14	No negative reactions in repeatedly treated cases	Decreased effusion, improved synovial score, improved histochemistry, no change in radiograph or MRI score as compared to control
Colbath et al. (97)	Bone marrow-derived MSCs	Chemically induced arthritis, *n* = 8	No difference in nucleated cell count between autologous and allogeneic MSCs	No improvement in clinical nor cytologic parameters
Magri et al. (82)	Umbilical cord-derived MSCs	Metacarpo- or metatarsophalangeal joint arthritis, *n* = 28	12% reported mild, transient heat or effusion	Significantly improved lameness and clinical score, 68% of horses back to athleticism
Delco et al. (83)	Adipose derived MSCs (a10^high^)	Tarsocrural impact model, *n* = 8	No	Significantly improved radiographic, gross, and histological score
Van Loon et al. (84)	Umbilical cord-derived MSCs	Clinical tendon and ligament injuries, *n* = 40	No	77% returned to equal or higher athleticism
Lange-Consiglio et al. (85)	Placenta-derived (*n* = 51) and bone marrow-derived MSCs (*n* = 44)	Clinical tendon and ligament injuries	No	4.00% of placenta derived and 23.08% of bone marrow-derived re-injured post treatment
Beerts et al. (90)	Peripheral blood-derived MSCs (tenogenic induced)	Clinical tendon and ligament injuries, *n* = 104	No	18% re-injury rate after 2 year follow-up

The use of bone marrow-derived allogeneic MSCs for soft tissue lesions show promise when the treatment is administered directly into the injured tissue. A clinical study of 40 horses treated with adipose-derived MSCs for tendon lesions concluded that 77% of those horses returned to full athletic function of equal or higher levels than prior to the injury (Table 2) (84). Another study using 44 clinical cases of tendon or ligament lesions showed a similar proportion of horses returning to athleticism after bone marrow-derived allogeneic MSC therapy (Table 2) (85). A recent large clinical equine study on soft tissue lesions found 18% of horses reinjuring within 2 years of follow up (Table 2) (86). These data appear favorable in comparison to the 44% re-injury rates among horses treated with rest and simple rehabilitation techniques alone (99).

When evaluating the therapeutic potential of allogeneic MSCs in experimental models of soft tissue lesions, laboratory animals were the only populations examined to date. Direct injection into a rat Achilles tendon rupture model results in improved elasticity and strength of treated tendons as compared to untreated tendons at 30 days post-treatment (100). Intrathecal injection of bone marrow-derived MSCs to treat a surgically created defect in the intra-synovial portion of the Achilles tendon in sheep does not improve healing of the treated tendons at 24 weeks post-injury (101). A study using adipose-derived allogeneic MSCs in a rat Achilles tendon tear model showed improved strength of the injured tendon when treated into the lesion with MSCs (102). Based on the evidence to date, tendons appear to have improved healing when treated with allogeneic MSCs, and the use of these treatments in equine tendon and ligament lesions is warranted.

## Repeated Allogeneic MSC Administration for Treatment of Disease

Few studies have been completed to determine if repeat administration of allogeneic MSCs is more beneficial than a single treatment. As we have detailed, there would likely be antibody presence in the animal upon repeat treatment along with memory Tcells (62, 71, 75). Repeat treatment using allogeneic MSCs has shown to cause an increase in leukocyte recruitment when used intra-articularly (32). One study using umbilical-derived MSCs showed no improvement in therapeutic efficacy when clinical cases of equine joint disease were treated twice in a 1 month interval as compared to those joints that were only treated once (82). As previously discussed, one rabbit study saw no improvement in arthritis when only one treatment of bone marrow-derived MSCs was given, while repeat therapy proved beneficial (98). In contrast, a study using mouse model of colitis showed that allogeneic MSCs improved the disease upon initial treatment, but when mice were again inflicted with colitis, only syngenic MSCs were beneficial, not the allogeneic MSCs that had provided therapy upon initial treatment (103). It is a common concern that repeat allogeneic therapy may lead to reduced therapeutic benefit in the horse, and we have yet to fully answer this question. Judging from the great amount of research showing immune response to interaction of MSCs and leukocytes, adaptive immunity likely will limit the functional ability of allogeneic MSCs upon repeat administration unless a means to mitigate MHC expression has been reconciled.

## Conclusion

Allogeneic MSCs have both immunostimulatory and immunosuppressive effects. Resounding immunosuppressive effects are seen when MSCs are mixed with activated neutrophils or activated lymphocytes (10, 11, 13, 28–31, 65). Allogeneic MSCs are recognized by the innate and adaptive arms of the immune system and their viability may be decreased following immune recognition (62, 64, 71). An antibody response is generated post-injection in the horse which likely would inhibit their therapeutic efficacy upon repeat treatment (32, 62, 64, 103). Allogeneic bone marrow- derived MSCs can survive in the recipient long term when delivered into low vascularity regions such as tendons and muscle (88, 90, 93).

There is evidence that use of allogeneic MSC therapy is beneficial to the patient (38, 81–86, 103). Results of several studies have shown allogeneic MSCs carry no greater rate of short-term complications when used as a one-off therapy as compared to other biologic therapies (8, 32–34), and improving laboratory techniques will continue to lower the occurrence of side effects (32). These side effects seen thus far have no relation to the level of success of the treatment (38, 40). The response generated from current allogeneic MSC therapies that may not survive long-term is substantial and should not be disregarded. Potentially, a more potent response will be generated from an MSC that is minimally recognized by the recipient immune system and allowed to have a longer time frame to exert a therapeutic effect (45). Methods to mitigate alloantibody production are being researched. ELA matching can be performed between recipient and donor. Molecular manipulation the MSCs to prevent expression of MHC I and II would decrease immune recognition. If repeat MSC therapy is given, variation of the donor MSC haplotype could minimize the immediate adaptive immune response. These options deserve continued investigation to improve upon the therapeutic benefits of allogeneic MSC therapy.

## Author Contributions

JLK constructed the manuscript. CR, NP, EG, and CWM edited the manuscript. All authors contributed to the article and approved the submitted version.

## Conflict of Interest

JLK and CWM are partners in Advanced Regenerative Therapies New Zealand. The remaining authors declare that the research was conducted in the absence of any commercial or financial relationships that could be construed as a potential conflict of interest.
